# Understanding Generation‐Z New Graduate Nurses' Intention to Stay: Applying the Stress and Coping Model

**DOI:** 10.1002/nop2.70520

**Published:** 2026-04-03

**Authors:** Euna Ju, Hyunjoo Na

**Affiliations:** ^1^ Seoul St. Mary's Hospital The Catholic University of Korea Seoul South Korea; ^2^ College of Nursing The Catholic University of Korea Seoul South Korea

**Keywords:** adaptation, nurses, nursing models, stress, work–life balance

## Abstract

**Aim:**

This study aimed to develop and evaluate a hypothetical model based on Lazarus and Folkman's stress and coping framework to identify the factors and pathways influencing Generation Z new graduate nurses' intention to stay in the job.

**Design:**

This model validation study employed a cross‐sectional path analysis approach using structural equation modelling.

**Methods:**

The participants were 215 nurses born after 1995 who had < 1 year of nursing experience and were working at three general hospitals located in Seoul, Korea. Structural equation modelling was used to analyse the collected data, and the model was validated through confirmatory factor analysis. Key variables included character strengths and job resources (exogenous) and reality shock, work–life balance and intention to stay (endogenous). Path analysis with phantom variables was conducted to explore mediating effects.

**Results:**

The model showed proper fit indices: *χ* ^2^/df = 2.34, TLI = 0.90, CFI = 0.91, RMSEA = 0.08, SRMR = 0.06. Character strengths (*ß* = 0.41, *p* < 0.001) and reality shock (*ß* = −0.75, *p* = 0.002) had significant direct effects, explaining 53.5% of the variance in intention to stay. Reality shock also mediated the relationships between character strengths, job resources and intention to stay, emphasising its pivotal role in retention strategies for Generation‐Z nurses. The findings indicate that character strengths and reality shock are important factors in the intention to stay of new graduate nurses in Generation‐Z.

**Patient or Public Contribution:**

No patient or public contribution.

## 1 Introduction

The World Health Organisation (WHO 2020) has estimated a global nursing shortage of 5.9 million. In South Korea, 35.3% of new nurses leave their jobs in hospitals within their first year for such reasons as poor job adaptation (Hospital Nurses Association 2023). The level of nursing staff is closely related to nursing‐sensitive patient outcomes (Blume et al. 2021), and thus, securing experienced nursing staff is crucial for ensuring patient safety in healthcare settings. Supporting new nurses in remaining in hospital positions, developing expertise and gaining clinical experience is essential to meeting these needs. However, many new nurses fail to adapt to the job and leave within the first year, making it is necessary to comprehensively determine the factors influencing their intention to stay in the job, including organisational characteristics and new nurses' personal attributes.

Members of Generation‐Z—those born after 1995—have recently entered the nursing workforce in hospitals. Having grown up in a digital environment, Generation‐Z is often characterised as ‘digital natives’ (Park 2019). In Korea, they are known as a generation that independently acquires needed information, values the present, prioritises materialistic values and is focused on consumption and leisure (Lee and Kim 2021; Park 2019). The most common reason for Generation‐Z new nurses leaving their jobs is poor job adaptation (36.5%) (Hospital Nurses Association 2023). Turnover within the first‐year increases when job autonomy, authority and opportunities for personal development are limited (Han 2022). This trend differs from previous generations of nurses, who often left their positions due to job dissatisfaction, lack of commitment to the organisation and negative interdisciplinary relationships (Vázquez‐Calatayud and Eseverri‐Azcoiti 2023). Therefore, to understand the factors influencing the intention to stay in the job among Generation‐Z new nurses, it is necessary to gain a differentiated understanding that reflects their values and characteristics.

## 2 Background

The transition of new nurses from students to professional practitioners as they begin working in hospitals often involves encountering discrepancies between expectations and reality. This adjustment period frequently results in value confusion, role conflicts and feelings of incompetence, collectively contributing to reality shock (Yun 2018). Previous studies have emphasised that reality shock is a significant factor influencing both turnover and intention to stay in the job (Go and Han 2020; Kim and Hyun 2022). Labrague and De Los Santos (2020) argued that to improve new nurses' intention to stay in the job, it is important to reduce reality shock and help them maintain a balance between professional life and personal life. Work–life balance refers to harmonious coexistence of work and other social activities, such as family, leisure, personal growth and self‐development, aligned with individual life priorities (Kim and Park 2008). The importance of work–life balance as a life value began to be emphasised by Generation‐Y, who typically defined as those born between 1981 and 1995 (Campbell and Patrician 2020); building on this balance, Generation‐Z has prioritised personal growth and job security (Sánchez‐Hernández et al. 2019). Previous studies have shown that the higher the work–life balance among nurses, the lower the turnover intention (Kwak and Kim 2021). Therefore, to understand factors influencing the intention to stay in the job among Generation‐Z new nurses, it is necessary to explore their experiences of reality shock and work–life balance and to examine the relationship between these factors.

According to Lazarus and Folkman's (1984) stress and coping theory, when faced with stressful situations, individuals assess how they perceive the stress event based on personal and environmental factors, which then leads to either adaptation or maladaptation through coping mechanisms. Even in the same situations and conditions, new nurses exhibit different levels of stress and responses depending on personality factors, which are a personal attribute (Sivaratnam et al. 2021). Among these personality factors, character strengths are positive personality traits reflected in emotions and behaviours, which everyone possesses (Peterson and Seligman 2004). Character strengths are typically conceptualised within the framework of Peterson and Seligman's Values in Action (VIA) classification, which delineates 24 strengths across six core virtues—wisdom, courage, humanity, justice, temperance and transcendence. Specific character strengths such as zest, perseverance, hope and curiosity foster healthy work behaviours and positive attitudes (Gander et al. 2012). Moreover, character strengths buffer stress (Sivaratnam et al. 2021), support coping and mental health (Harzer and Ruch 2015; Casali et al. 2021) and are associated with reduced turnover intention among nurses (Chu et al. 2022). Therefore, it is necessary to examine whether Generation‐Z new nurses, who prioritise personal growth, are influenced in their intention to stay in the job by their ability to overcome stressful situations.

Job resources can be considered an environmental factor influencing nurses' intention to stay. Previous studies have shown that when the nursing work environment is good (Holland et al. 2019; McClain et al. 2022) and when social support from supervisors or colleagues is strong (McClain et al. 2022; Yu et al. 2021), the intention to stay is higher owing to the abundance of job resources. Job resources are organisational resources that can reduce job stress; they include job autonomy, social support, organisational support, adequacy of compensation and organisational fairness (Occupational Safety and Health Research Institute 2019). New nurses who perceive their nursing work environment as poor are shown to experience higher levels of reality shock (Moon and Cho 2022) and are related to both turnover intention and intention to stay (Scanlan and Still 2019; Yu et al. 2021). Therefore, it is necessary to comprehensively examine the impact of personal factors, such as character strengths, and environmental factors, such as job resources, on the intention to stay.

Previous studies that constructed theoretical models of nurses' intention to stay have mostly investigated the causal relationships of variables reflecting organisational characteristics (Cho et al. 2021; Cowden and Cummings 2015). Few studies have considered generational characteristics, including personality and values, as influencing factors. Against this backdrop, this study aims to construct a conceptual framework based on Lazarus and Folkman's (1984) stress and coping theory to identify a predictive model influencing the intention to stay in the job.

### 2.1 Conceptual Framework of the Study

**FIGURE 1:**
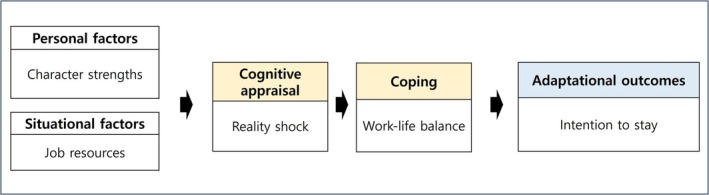
Conceptual framework of the study.

This study constructed the theoretical framework shown in Figure 1 to build a model predicting the intention to stay among Generation‐Z new nurses, based on Lazarus and Folkman's (1984) stress and coping theory and a review of previous literature. This study assumes that new nurses, as newcomers to the workforce, encounter difficulties in job adaptation, and their adaptive outcomes may differ depending on the level of environmental resources they possess, as well as their cognitive appraisal and coping mechanisms. In this model, personal and situational factors are represented by character strengths and job resources, which function as resources that determine the stress experienced.

Cognitive appraisal is represented by reality shock, defined as the process by which individuals interpret or evaluate a life event cognitively, determining whether the event acts as a stressor for them. It involves a primary appraisal of whether an event is threatening and a secondary appraisal of how the individuals can respond to the perceived threat. New nurses experience reality shock during the process of job adaptation, and it is necessary to examine whether personal and situational factors influence this process and affect their intention to stay.

Coping is the process by which individuals continuously alter their cognition and behaviour to manage stress when they experience it. Work–life balance is an overall assessment of the balance between work, family, leisure and personal growth and indicates what Generation‐Z prioritises and acts upon to cope with stress.

Adaptation, as the outcome of appraisal and coping, includes both positive and negative effects, ranging from immediate emotions to long‐term well‐being. In this study, the intention to stay in the job is explained as an outcome resulting from the interaction of all these factors, whereby new nurses experience reality shock (cognitive appraisal) in response to personal and situational resources and seek work–life balance (coping) to resolve the situation.

The study model sets character strengths as personal factors, job resources as situational factors, reality shock as the cognitive appraisal from the interaction between the individual and the situational, work–life balance as the coping mechanism and intention to stay as the adaptational outcome of the stress response. Finally, a model influencing the intention to stay is established, and the direct, indirect and mediating effects between variables are identified.

## 3 Methods

### 3.1 Design

This model validation study, grounded in Lazarus and Folkman's (1984) stress and coping theory, employed a cross‐sectional path analysis approach using structural equation modelling. This study constructed a hypothetical model of factors influencing Generation‐Z new nurses' intention to stay in the job, verified the model's fit and analysed the causal and mediating relationships among the variables proposed variables (Figure 1).

### 3.2 Study Participants

The study participants were Generation‐Z new nurses working at one advanced general hospital and two general hospitals in Seoul, South Korea. In South Korea, registered nurses typically graduate from a 4‐year bachelor's degree programme at a nursing school. The selection criteria were nurses born after 1995, those who had obtained their first job in a hospital after graduating from nursing school, those who had completed preceptorship and were independently performing their duties, those with < 1 year of clinical experience, and those who understood the purpose and content of the study and agreed to participate.

A convenience sampling method was used to recruit Generation‐Z new nurses. A minimum of 10 samples is required per observed variable (Mitchell 2020). As this study has 19 observed variables, the minimum recommended sample size is 190. If using maximum likelihood estimation, a typical method in structural equation modelling, at least 200 participants are needed (Woo 2022). Assuming 200 participants and considering an approximate dropout rate of 25% owing to the online survey method, the sample size was set to at least 250. There were 256 survey respondents in total 256. Of these, seven were excluded for not meeting the selection criteria, and 34 for providing insincere responses, leaving 215 respondents for the final analysis.

### 3.3 Instruments

#### 3.3.1 Character Strengths

Character strengths were measured using the Korean version of the Value in Action Inventory of Strengths (VIA‐IS) developed by Peterson and Seligman (2004) and shortened to 48 items by McGrath (2019). VIA‐IS assessed 24 strengths grouped under six virtues: wisdom and knowledge, courage, humanity, justice, temperance and transcendence. Each item is scored on a 5‐point Likert scale (from 1 ‘very much unlike me’ to 5 ‘very much like me’), with higher scores indicating higher levels of character strengths. Sixteen items were reverse scored. McGrath (2019) reported a Cronbach's *α* of 0.80, whereas the reliability in this study was 0.90.

#### 3.3.2 Job Resources

Job resources were measured using a tool developed by the Occupational Safety and Health Research Institute (2019), which was tested for reliability and validity among Korean nurses. This tool consists of 18 items across five sub‐factors: job autonomy, social support, organisational support, adequacy of compensation and organisational fairness. Each item is scored on a 4‐point Likert scale (from 1 ‘Strongly disagree’ to 4 ‘strongly agree’), with higher scores indicating greater job resources. The original instrument (Occupational Safety and Health Research Institute 2019) reported a Cronbach's *α* of 0.93, whereas the reliability in this study was 0.89.

#### 3.3.3 Reality Shock

Reality shock was measured using a tool developed by Yun (2018) to assess the reality shock of new nurses. This tool consists of 26 items across five sub‐factors: disappointment with reality, feeling overwhelmed at work, lack of confidence, excessive responsibility and lack of support. Four items related to lack of support were excluded because they overlapped conceptually with the sub‐factor of social support under job resources, leaving 22 items for final use. Each item is scored on a 5‐point Likert scale (from 1 ‘Strongly disagree’ to 5 ‘strongly agree’), with higher scores indicating greater reality shock experienced by new nurses. Yun (2018) reported a Cronbach's *α* of 0.93, and the reliability in this study was also 0.93.

#### 3.3.4 Work–Life Balance

Work–life balance was measured using a tool developed by Kim and Park (2008). This tool consists of 29 items across four sub‐factors: work–family balance, work–leisure balance, work–growth balance and overall evaluation. In this study, to assess the impact of each aspect of life balance on work and life, the four items from the overall evaluation were excluded, leaving 25 items for final use. Each item is measured on a 5‐point Likert scale (from 1 ‘Strongly disagree’ to 5 ‘strongly agree’), and as the items are phrased to assess imbalance, all items were reverse‐scored. Higher scores indicate that work–life balance is being positively maintained. Kim and Park (2008) reported a Cronbach's *α* of 0.93, whereas the reliability in this study was 0.94.

#### 3.3.5 Intention to Stay

Intention to stay is the intention to continue your nursing career rather than leave for another job and was measured using a tool adapted by Kim (2006) from Cowin's (2002) Nurses' Retention Index. This tool consists of six items, scored on an 8‐point Likert scale (from 1 ‘Strongly disagree’ to 8 ‘strongly agree’), with higher scores indicating a stronger intention to stay among new nurses. Two negatively worded items were reverse scored. The reliability (Cronbach's *α*) was 0.97 in the original development study (Cowin 2002), 0.89 in the Korean version (Kim 2006) and 0.95 in this study.

### 3.4 Data Collection

The research team visited the nursing departments of the three hospitals to explain the study's purpose and methods, and obtained prior approval. The survey was conducted online during September 2022. After receiving approval from the Institutional Review Board (IRB), cooperation for data collection was sought from the nursing departments of each hospital. The researcher distributed recruitment notices to the nursing departments and nursing managers. New nurses who wished to participate in the survey voluntarily could access the Google Forms URL by scanning the QR (quick response) code provided in the recruitment notice.

### 3.5 Data Analysis

The collected data were analysed using IBM SPSS 26.0 and AMOS 26.0. The general characteristics of the participants were analysed using frequency, percentage, mean and standard deviation. Differences in research variables according to general characteristics were analysed using *t*‐tests and ANOVA. Pearson's correlation coefficient was used to analyse the correlations among character strengths, job resources, reality shock, work–life balance and intention to stay. Normality of the research variables was verified by examining skewness and kurtosis values and multicollinearity was checked by calculating correlation coefficients, tolerance and variance inflation factor (VIF) values. The validity of the structural model predicting Generation‐Z new nurses' intention to stay was verified using confirmatory factor analysis and tested through construct reliability and average variance extracted (AVE). To assess the model fit of the hypothesised model, the maximum likelihood method was used. Absolute fit indices, including *χ* ^2^, df, *χ* ^2^/df, root mean‐square error of approximation (RMSEA), and standardised root mean‐square residual (SRMR), were used, as well as incremental fit indices, including the Tucker–Lewis index (TLI) and comparative fit index (CFI). To verify the statistical significance of indirect and total effects in the model, the AMOS bootstrapping method was performed 1000 times, and effects were tested at a 95% confidence interval (CI). The AMOS phantom variable method was used to analyse the dual mediating effects of reality shock and work–life balance.

## 4 Results

### 4.1 Participants' Characteristics

A total of 215 participants were included in this study, with 202 (94.0%) being female, and the average age was 23.8 years. The majority, 173 (80.5%), were aged 22–24 years, born between 1998 and 2000, while 42 (19.5%) were aged 25–27 years, born between 1995 and 1997. The average clinical experience of the participants was 6.6 months, and the largest number, 65 (30.2%), worked in medical wards. There were no significant differences in intention to stay in the job based on the general characteristics of the study participants (Table 1).

**TABLE 1:** Difference in intension to stay according to general characteristics (*N* = 215).

Variables	Categories	*n* (%)	*M* ± SD	Intension to stay	
				*M* ± SD	*t* or *F* (*p*)
Gender	Woman	202 (94.0)		4.70 ± 1.69	−0.03 (0.977)
	Man	13 (6.0)		4.72 ± 2.05	
Age (year)	22~24	173 (80.5)	23.83 ± 1.08	4.69 ± 1.68	−0.34 (0.733)
	25~27	42 (19.5)		4.79 ± 1.84	
Length of time working in hospital (months)	2~5	83 (38.6)	6.59 ± 2.78	5.03 ± 1.77	2.45 (0.088)
	6~9	85 (39.5)		4.50 ± 1.71	
	10~12	47 (21.9)		4.50 ± 1.55	
Department	Medical ward	65 (30.2)		4.51 ± 1.69	1.31 (0.269)
	Surgical ward	33 (15.3)		4.75 ± 1.70	
	CNCS ward	31 (14.4)		4.54 ± 1.68	
	ICU	42 (19.5)		4.56 ± 1.77	
	Special unit	44 (20.5)		5.20 ± 1.69	

*Note:* Special unit, operation room, emergency room, delivery room and Hospice ward.

Abbreviations: CNCS, comprehensive nursing care services; ICU, intensive care unit.

### 4.2 Descriptive Statistics for Measurement Variables

The descriptive statistics for the measurement variables are shown in Table 2. The mean scores for the participants were as follows: character strengths, 3.42 ± 0.37; job resources, 2.69 ± 0.38; work–life balance, 2.78 ± 0.69; and intention to stay, 4.70 ± 1.71. The skewness of the study variables ranged from −0.39 to 0.92, and kurtosis ranged from −0.49 to 0.73, none of which exceeded ±1.965, indicating a normal distribution at the 0.05 significance level. The multivariate kurtosis was 6.952, and the critical ratio was 1.904, which did not exceed ±1.965, thus confirming multivariate normality. Multicollinearity among the independent variables was assessed with a correlation coefficient (*r*) < 0.80, tolerance > 0.1, and VIF < 10 (Shrestha 2020). In this study, correlation coefficients ranged from −0.71 to 0.56, tolerance from 0.39 to 0.71, and VIF from 1.41 to 2.56, indicating no issues with multicollinearity among the independent variables.

**TABLE 2:** Descriptive statistics and validity of variables (*N* = 215).

Latent variables	Measured variables	Range	Min	Max	*M* ± SD	AVE	C.R.
Character strengths	Humanity	1–5	2.25	5.00	3.73 ± 0.54	0.75	0.95
	Justice	1–5	2.13	4.75	3.52 ± 0.44		
	Temperance	1–5	2.00	4.63	3.50 ± 0.49		
	Transcendence	1–5	1.75	4.75	3.44 ± 0.56		
	Wisdom and knowledge	1–5	1.75	4.38	3.19 ± 0.54		
	Courage	1–5	1.75	4.38	3.14 ± 0.52		
	Total character strengths	1–5	2.50	4.48	3.42 ± 0.37		
Job resources	Social support	1–4	1.67	4.00	3.26 ± 0.46	0.83	0.96
	Job autonomy	1–4	1.33	4.00	2.80 ± 0.41		
	Organisational fairness	1–4	1.25	4.00	2.66 ± 0.45		
	Organisation support	1–4	1.20	4.00	2.62 ± 0.49		
	Adequacy of compensation	1–4	1.00	4.00	2.13 ± 0.59		
	Total job resources	1–4	1.39	3.72	2.69 ± 0.38		
Reality shock	Overwhelming task	1–5	1.00	5.00	3.59 ± 0.82	0.81	0.94
	Lack of confidence	1–5	1.33	5.00	3.52 ± 0.77		
	Disappointment to reality	1–5	1.00	5.00	3.26 ± 0.71		
	Heavy responsibility	1–5	1.00	5.00	3.23 ± 0.79		
	Total reality shock	1–5	1.23	4.82	3.42 ± 0.65		
Work‐life balance	Work‐family balance	1–5	1.38	5.00	3.10 ± 0.72	0.77	0.91
	Work‐leisure balance	1–5	1.00	5.00	2.64 ± 0.87		
	Work‐growth balance	1–5	1.11	5.00	2.63 ± 0.75		
	Total work‐life balance	1–5	1.28	4.84	2.78 ± 0.69		
Intension to stay		1–8	1.00	8.00	4.70 ± 1.71		

Abbreviations: AVE, average variance extracted; C.R., composite reliability.

### 4.3 Correlations Between Measurement Variables

Reality shock showed a significant negative correlation with character strengths (*r* = −0.49, *p* < 0.001), job resources (*r* = −0.65, *p* < 0.001), work–life balance (*r* = −0.71, *p* < 0.001) and intention to stay (*r* = −0.59, *p* < 0.001). Work–life balance showed a significant positive correlation with character strengths (*r* = 0.48, *p* < 0.001), job resources (*r* = 0.56, *p* < 0.001) and intention to stay (*r* = 0.40, *p* < 0.001). Intention to stay showed a significant positive correlation with character strengths (*r* = 0.52, *p* < 0.001) and job resources (*r* = 0.52, *p* < 0.001) (Table 3).

**TABLE 3:** Correlations between measurement variables (*N* = 215).

Variables	*r* (*p*)				
	1	2	3	4	5
1. Character strengths	1				
2. Job resources	0.44 (< 0.001)	1			
3. Reality shock	−0.49 (< 0.001)	−0.65 (< 0.001)	1		
4. Work‐life balance	0.48 (< 0.001)	0.56 (< 0.001)	−0.71 (< 0.001)	1	
5. Intension to stay	0.52 (< 0.001)	0.52 (< 0.001)	−0.59 (< 0.001)	0.40 (< 0.001)	1

### 4.4 Validity Assessment of the Hypothesised Model

**FIGURE 2:**
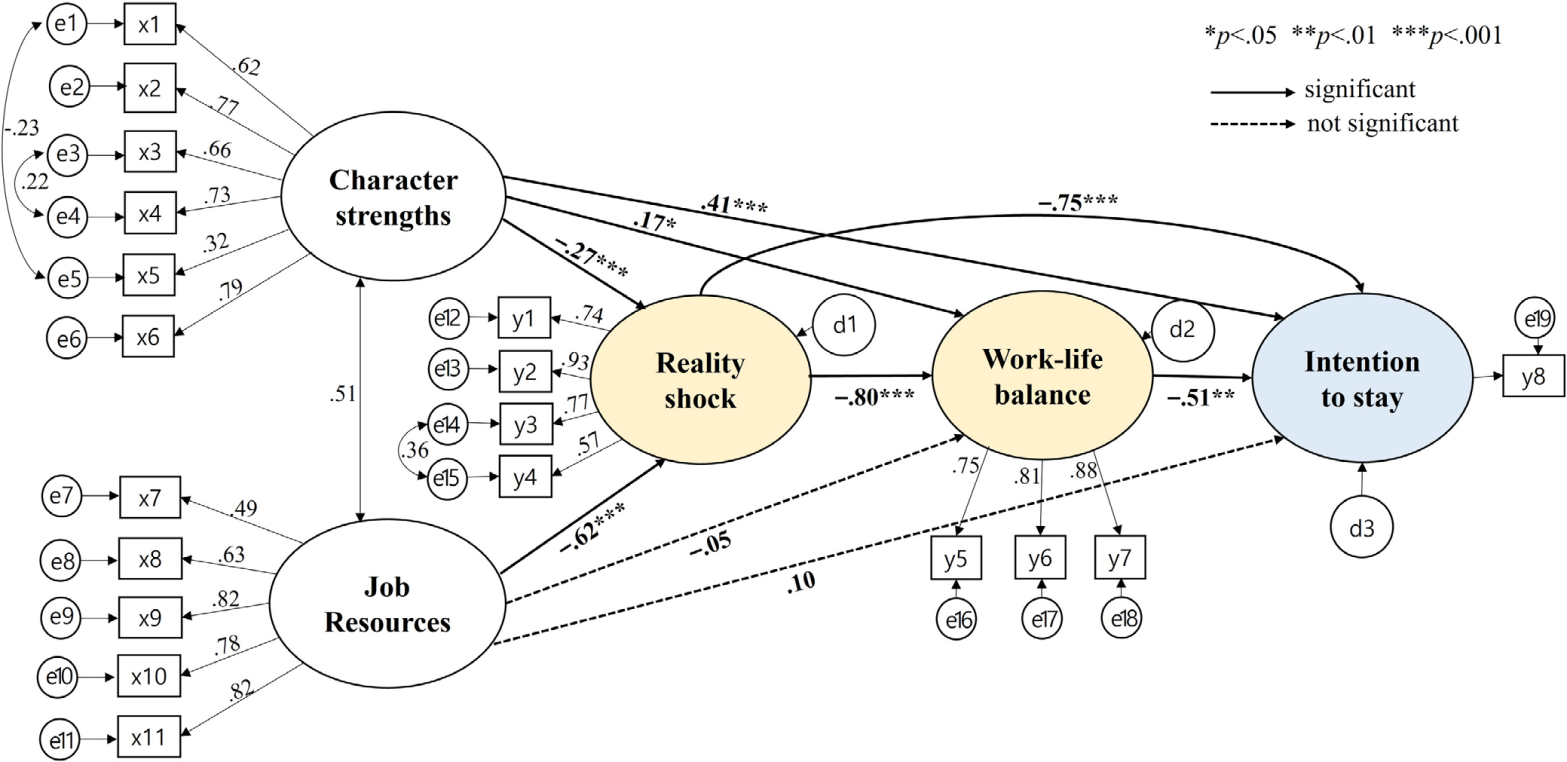
The final model of intention to stay among new graduate nurses in Generation‐Z. x1, wisdom and knowledge; x2, courage; x3, humanity; x4, justice; x5, temperance; x6, transcendence; x7, job autonomy; x8, social support; x9, organisation support; x10, adequacy of compensation; x11, organisational fairness; y1, disappointment to reality; y2, overwhelming task; y3, lack of confidence; y4, heavy responsibility; y5, work‐family balance; y6, work‐leisure balance; y7, work‐growth balance; y8, intention to stay.

To verify the convergent validity of the latent variables, this study checked whether the standardised coefficients (*β*) were above 0.50, the AVE above 0.50 and the composite reliability above 0.70 (Figure 2, Table 2). The standardised coefficients ranged from 0.32 to 0.93, with temperance, a sub‐factor of character strengths, at 0.32, and job autonomy, a sub‐factor of job resources, at 0.49, both below the criterion. The standardised coefficients should be at least 0.30 (Woo 2022). In this study, the coefficients were above the minimum criterion and the model fit was adequate; thus, no items were removed, as their exclusion could alter the conceptual meaning of the constructs. The AVE for the latent variables—character strengths, job resources, reality shock and work–life balance—ranged from 0.75 to 0.83, and composite reliability ranged from 0.91 to 0.96, meeting the criteria and confirming the convergent validity of the latent variables. Discriminant validity, which checks whether the constructs are distinct from each other, was confirmed by verifying that the AVE for the latent variables was greater than the squared correlations between them. The AVE ranged from 0.75 to 0.83, and the squared correlations from 0.20 to 0.74, confirming discriminant validity.

### 4.5 Fit Assessment of the Hypothesised Model

The fit of the hypothesised model was judged to be acceptable if *χ* ^2^/df was below 3.0, RMSEA below 0.10, SRMR below 0.10, CFI above 0.90 and TLI above 0.90 (Woo 2022). The fit indices for the measurement model were *χ* ^2^/df = 2.60, RMSEA = 0.09, SRMR = 0.06, CFI = 0.90, which were satisfactory. However, the TLI was slightly below the criterion at 0.88, and thus, covariance relationships within the acceptable range were added using modification indices (MI). Covariance relationships were added between the measurement errors of lack of confidence and excessive responsibility within reality shock (MI = 24.04), between wisdom and knowledge and temperance within character strengths (MI = 9.01), and between humanity and justice within character strengths (MI = 6.90). The fit indices for the modified model were *χ* ^2^/df = 2.34, RMSEA = 0.08, SRMR = 0.06, CFI = 0.91 and TLI = 0.90, all meeting recommended levels. Therefore, the modified model was chosen as the final model (Figure 2).

### 4.6 Verification of Direct, Indirect and Mediating Effects Using Path Analysis

An effect analysis was conducted to determine the direct and indirect effects among the latent variables related to Generation‐Z new nurses' intention to stay, and significance was verified through bootstrapping (Table 4). For intention to stay, character strengths showed significant direct effects (*ß* = 0.41, *p* = 0.002) and total effects (*ß* = 0.41, *p* = 0.002), while job resources showed significant indirect effects (*ß* = 0.24, *p* = 0.005) and total effects (*ß* = 0.34, *p* = 0.002). Reality shock showed significant direct effects (*ß* = −0.75, *p* = 0.004), indirect effects (*ß* = 0.41, *p* = 0.004) and total effects (*ß* = −0.34, *p* = 0.005) on intention to stay, while work–life balance showed significant direct effects (*ß* = −0.51, *p* = 0.004). The explanatory power of the predictive variables for intention to stay was 53.5%.

**TABLE 4:** Standardised direct, indirect and total effects for the modified model (*N* = 215).

Endogenous variables	Exogenous variables	*B*	SE	CR (*p*)	SMC	Direct effect *ß* (*p*)	Indirect effect *ß* (*p*)	Total effect *ß* (*p*)
Reality shock	Character strengths	−0.46	0.12	−3.71 (< 0.001)	0.622	−0.27 (0.002)		−0.27 (0.002)
	Job resources	−0.69	0.10	−7.30 (< 0.001)		−0.62 (0.002)		−0.62 (0.002)
Work‐life balance	Character strengths	0.37	0.15	2.50 (0.012)	0.760	0.17 (0.042)	0.22 (0.002)	0.39 (0.003)
	Job resources	−0.07	0.13	−0.55 (0.581)		−0.05 (0.689)	0.49 (0.002)	0.44 (0.002)
	Reality shock	−1.01	0.14	−7.17 (< 0.001)		−0.80 (0.003)		−0.80 (0.003)
Intention to stay	Character strengths	2.24	0.48	4.72 (< 0.001)	0.535	0.41 (0.002)	0.01 (0.907)	0.41 (0.00 2)
	Job resources	0.38	0.38	1.00 (0.317)		0.10 (0.321)	0.24 (0.005)	0.34 (0.002)
	Reality shock	−2.44	0.63	−3.85 (< 0.001)		−0.75 (0.004)	0.41 (0.004)	−0.34 (0.005)
	Work‐life balance	−1.31	0.43	−3.05 (0.002)		−0.51 (0.004)		−0.51 (0.004)

Abbreviations: *B*, estimate; CR, critical ratio; SE, standard error; SMC, squared multiple correlation; *ß*, standardised estimate.

The hypothesised model in this study is a dual mediation model. Bootstrapping was conducted using phantom variables to analyse the significance of individual indirect effects along each mediation pathway. The results of the dual mediation analysis confirmed that the pathways from character strengths → reality shock → work–life balance → intention to stay (*B* = −0.61, 95% CI = [−1.521~−0.231]) and from job resources → reality shock → work–life balance → intention to stay (*B* = −0.91, 95% CI = [−2.314~−0.343]) were both significant (Table 5, Figure 2).

**TABLE 5:** Path analysis result of individual mediating effects using phantom variables (*N* = 215).

Pathway	*B*	SE	95% CI		*p*
			Lower	Upper	
Character strengths → RS → Work‐life balance	0.46	0.14	0.221	0.766	0.002
Character strengths → RS → Intention to stay	1.12	0.52	0.466	2.404	0.002
Character strengths → WLB → Intention to stay	−0.48	0.34	−1.379	−0.037	0.030
Character strengths → RS → WLB → Intention to stay	−0.61	0.37	−1.521	−0.231	0.003
Job resources → RS → Work‐life balance	0.70	0.15	0.456	1.110	0.001
Job resources → RS → Intention to stay	1.69	0.67	0.789	3.195	0.003
Job resources → WLB → Intention to stay	0.09	0.29	−0.268	0.860	0.639
Job resources → RS → WLB → Intention to stay	−0.91	0.53	−2.314	−0.343	0.003

Abbreviations: *B*, estimate; CI, confidence interval; RS, reality shock; SE, standard error; WLB, work‐life balance.

## 5 Discussion

This study constructed a model predicting Generation‐Z new nurses' intention to stay based on Lazarus and Folkman's (1984) stress and coping theory and validated the model by analysing the causal relationships among the variables. The results showed that character strengths, job resources, reality shock and work–life balance all had significant direct effects on Generation‐Z new nurses' intention to stay, with reality shock being the most influential factor. To further investigate the indirect effects, a dual mediation analysis using phantom variables confirmed that both character strengths and job resources significantly influenced intention to stay through the sequential mediating effect of reality shock and work–life balance. The explanatory power for Generation‐Z new nurses' intention to stay was 53.5%, and the model fit met recommended levels, indicating that the study model is suitable for explaining the intention to stay among Generation‐Z new nurses.

In this study, character strengths, as a personal factor, had significant direct effects on reality shock, work–life balance and intention to stay. Generation‐Z new nurses with higher levels of character strengths experienced lower levels of reality shock, which is consistent with Zhang et al. (2021), who found that higher levels of character strengths were associated with lower psychological stress. Higher levels of character strengths were also associated with higher work–life balance and intention to stay, aligning with previous findings that character strengths reduce turnover intention (Chu et al. 2022). Overall, it can be understood that when new nurses face stressful situations upon joining a hospital, those with higher levels of character strengths experience less reality shock, adapt better and are more likely to stay. Having high levels of character strengths means utilising more positive resources in stressful situations, which can reduce the stress response (Sivaratnam et al. 2021). Gander et al. (2012) found that among the 24 character strengths, attributes such as hope, zest and courage are particularly important in facilitating active coping behaviours. According to a meta‐analysis by Schutte and Malouff (2019), interventions that enhance individuals' signature strengths reduce depression and increase happiness and life satisfaction, yielding positive effects across various life domains. These outcomes are possible because character strengths are not fixed traits but can be cultivated through sustained practice and interventions. Training programs that help individuals identify three to seven signature strengths that uniquely represent them and intentionally apply these strengths in daily life and professional practice have been shown to further promote these benefits. Therefore, to increase Generation‐Z new nurses' intention to stay, it is necessary to develop and implement intervention and educational programmes that utilise character strengths.

The study results showed that job resources, a situational factor, had a significantly greater direct impact on the reality shock experienced by Generation‐Z new nurses than did character strengths, a personal factor. This finding is consistent with the argument of Masso et al. (2022), who suggested that, rather than focusing on enhancing the competencies of new nurses, it is crucial to assess whether the organisation provides the resources necessary to support their growth and development to reduce reality shock. Therefore, to reduce the reality shock experienced by Generation‐Z new nurses, it is necessary to explore ways to enhance job resources, including job autonomy, social support, organisational support, adequate compensation and organisational fairness.

Moreover, the structural equation model results showed that job resources did not have a direct impact on intention to stay but rather influenced intention to stay indirectly through the mediation of reality shock. Previous studies found that the nursing work environment directly influenced turnover intention (Kwak and Kim 2021; Lee et al. 2022) and that job resources were related to nurses' burnout, job satisfaction and turnover intention (Scanlan and Still 2019), which contrasts with the findings of this study. However, the results of this study are consistent with those that reported the nursing work environment influences turnover intention by mediating psychological stress (Lee et al. 2022). It can be inferred that higher job resources among Generation‐Z new nurses lead to less reality shock, which in turn increases their intention to stay.

In this study, reality shock had a direct effect on both work–life balance and intention to stay, and it was identified as the most significant factor directly influencing intention to stay. Furthermore, reality shock showed significant mediating effects in the relationships between character strengths and work–life balance, character strengths and intention to stay, job resources and work–life balance, and job resources and intention to stay. This is consistent with previous studies that found lower reality shock among new nurses was associated with higher intention to stay (Kim and Hyun 2022), and that reality shock was related to new nurses' turnover intention (Go and Han 2020). Because Generation‐Z new nurses experience high levels of reality shock, and reality shock has the greatest impact on their intention to stay, strategies to reduce reality shock are crucial for increasing their intention to stay.

The Generation‐Z new nurses in this study experienced the highest levels of the sub‐factor overwhelming task, feeling burdened by an excessive workload within the reality shock construct. Kim et al. (2024) compared the work values of Generation‐Y and Generation‐Z new nurses, and found that task value, which includes task difficulty and workload, had a positive effect on job satisfaction for Generation‐Y but a negative effect for Generation‐Z, highlighting generational differences. Generation‐Y new nurses experienced a sense of achievement and job satisfaction from completing difficult tasks, whereas Generation‐Z new nurses found that tasks that were too challenging for their abilities negatively impacted their job satisfaction. Therefore, to prevent reality shock in Generation‐Z new nurses, it is particularly important to develop specific policies that reduce the number of patients they care for, allow them to learn easier tasks at a slower pace, and ensure that staffing levels match the workload of the department.

Meanwhile, this study found that reality shock and work–life balance had significant dual mediating effects on the pathway from character strengths and job resources to intention to stay. Although the correlation analysis showed a significant positive correlation between work–life balance and intention to stay, the structural equation model results indicated that work–life balance had a negative direct effect on intention to stay, making careful interpretation of the results necessary. When the sign of the correlation coefficient between two variables is opposite to the sign of the path coefficient in the model, a suppression effect may be present (Cohen et al. 2003). Additionally, in the analysis of the mediating effect of work–life balance on the relationship between reality shock and intention to stay, reality shock showed a negative direct effect, a positive indirect effect and a reduced negative total effect on intention to stay, which can be interpreted as a suppression effect of work–life balance on the relationship between reality shock and intention to stay (Martinez Gutierrez and Cribbie 2021). The suppression effect of work–life balance on the relationship between reality shock and intention to stay can be interpreted to mean that even if new nurses experience high reality shock, maintaining a healthy work–life balance can lessen the decrease in their intention to stay.

The mediating effect of work–life balance in suppressing reality shock is like a previous finding that work–life balance positively impacted job stress reduction and psychological well‐being issues, like burnout, among nurses (Xiao et al. 2023). While Generation‐Y new nurses tend to focus more on the job itself, Generation‐Z new nurses places higher importance on personal life and leisure, prioritising a healthy work–life balance (Kim et al. 2024). Therefore, to help new nurses manage and cope with the stress they experience when starting at a hospital, strategies to maintain work–life balance are needed. It is essential to secure appropriate work schedules and leave that allow for personal time for leisure activities or exercise, as well as systematic support for nurses to manage their careers and foster personal growth.

### 5.1 Study Limitations and Strengths

The data were collected using convenience sampling from new nurses at three advanced general hospitals and general hospitals in Seoul, South Korea, which limits the generalisability of the results to all new nurses. As this study is a cross‐sectional survey, it is not possible to definitively conclude that the statistical causal relationships between variables reflect actual phenomena. All measures were based on self‐report instruments, which may be subject to social desirability bias and response distortion. The suppression effect of work–life balance in the relationship between reality shock and intention to stay was identified through the structural equation model, which allowed for the simultaneous estimation of multiple relationships among variables. While this provided a deeper understanding of the mediating effect of work–life balance among Generation‐Z new nurses, the findings are from a single study and cannot be generalised without further replication. Although the sample size met methodological recommendations for SEM (Mitchell 2020; Woo 2022), it did not reach the commonly cited threshold of 300 cases. Therefore, the generalisability of the findings may be limited, and future studies should seek to test the model using larger and more diverse samples.

Nonetheless, this study is significant in that it examined the organic pathways influencing Generation‐Z new nurses' intention to stay based on Lazarus and Folkman's (1984) stress and coping theory, thereby building on previous research and presenting causal relationships among variables in greater detail. The intention to stay among Generation‐Z new nurses was found to be significantly influenced by character strengths and job resources, mediated by reality shock, with each variable having a statistically significant impact on intention to stay. These findings contribute to a better understanding of the factors influencing Generation‐Z new nurses' intention to stay in the job and provide a theoretical basis for developing policies to increase intention to stay among new nurses.

Our analysis focused on testing a conceptual model. Future research should longitudinally investigate how reality shock, work–life balance and intention to stay change over time as new nurses gain experience. Furthermore, additional variables that reflect Generation‐Z characteristics should be explored in our model beyond character strengths and job resources with regard to influencing intention to stay in the job among Generation‐Z new nurses.

### 5.2 Implications for Practice and Policy

As reality shock was found to be the most significant factor directly affecting intention to stay, efforts at the nursing school or hospital level alone are insufficient; institutional support at the national level is required to reduce reality shock among new nurses. Several literature reviews have reported that nurse residency programs, designed to help new nurses adapt clinically, have positive effects on confidence, practical competency, job satisfaction and intention to stay (Masso et al. 2022). Although recent reports indicate that the Korean Nurse Residency Program significantly reduces turnover rates and intention to leave within the first year (Song et al. 2024), it is currently implemented in only a few medical institutions in Korea. It is necessary to institutionalise and expand the programme nationwide. Additionally, to reduce reality shock and increase intention to stay, it is important to explore organisational strategies to enhance job resources, including job autonomy, social support, organisational support, adequate compensation and organisational fairness. Developing and implementing intervention programs that enhance the character strengths of Generation‐Z new nurses is also necessary.

## 6 Conclusions

This study constructed and validated a model predicting the intention to stay in the job among Generation‐Z new nurses based on stress and coping theory and findings from previous research. The results showed that character strengths and reality shock had direct effects on intention to stay. The study found that the personal factor of character strengths and the environmental factor of job resources influenced the intention to stay through the mediating effects of reality shock and work–life balance. Based on the results, this study concludes that it is necessary to develop intervention programs to develop the character strengths of new nurses, ensure adequate job resources to reduce reality shock and establish national‐level institutional policies to maintain work–life balance.

## Author Contributions

Conceptualisation or/and methodology: E.J. and H.N. Data curation or/and formal analysis: E.J. Funding acquisition: H.N. Investigation: E.J. Project administration or/and supervision: E.J. and H.N. Resources or/and software: E.J. Validation: E.J. and H.N. Visualisation: E.J. Writing: original draft or/and review and editing: E.J. and H.N.

## Funding

This study was supported by the National Research Foundation of Korea (No. NRF‐2022R1A2C1011568).

## Disclosure

The statistics were checked prior to submission by an expert statistician: Lee Il‐Hyun, tarra@statedu.com, PhD in Statistics.

## Ethics Statement

Ethical approval was granted by the St' Mary's Hospital, the Catholic University of Korea (IRB no: XC22QIDI0070). Informed consent was obtained from all participants prior to participation.

## Conflicts of Interest

The authors declare no conflicts of interest.

## Data Availability

The data supporting the findings of this study are available from the corresponding author upon reasonable request.
